# The Protective Effect of Polyphenols for Colorectal Cancer

**DOI:** 10.3389/fimmu.2020.01407

**Published:** 2020-07-10

**Authors:** Sujuan Ding, Sheng Xu, Jun Fang, Hongmei Jiang

**Affiliations:** Hunan Provincial Engineering Research Center of Applied Microbial Resources Development for Livestock and Poultry, College of Bioscience and Biotechnology, Hunan Agricultural University, Changsha, China

**Keywords:** polyphenols, colorectal cancer, intestinal inflammation, epigenetics, microbe

## Abstract

Colorectal cancer (CRC) is one of the most prevalent cancers that threaten people in many countries. It is a multi-factorial chronic disease caused by a combination of genetic and environmental factors, but it is mainly related to lifestyle factors, including diet. Plentiful plant foods and beverages are abundant in polyphenols with antioxidant, anti-atherosclerotic, anti-inflammatory, and anticancer properties. These compounds participate in host nutrition and disease pathology regulation in different ways. Polyphenolic compounds have been used to prevent and inhibit the development and prognosis of cancer, and examples include green tea polyphenol (–)epigallocatechin-3-O-gallate (EGCG), curcumin, and resveratrol. Of course, there are more known and unknown polyphenol compounds that need to be further explored for their anticancer properties. This article focuses on the fact that polyphenols affect the progression of CRC by controlling intestinal inflammation, epigenetics, and the intestinal microbe in the aspects of prevention, treatment, and prognosis.

## Introduction

Colorectal cancer (CRC) is one of the most common malignancies worldwide (1, 2). Like many diseases, the formation of CRC is caused by various genetic and environmental factors (3). Globally in 2018, CRC ranked third in the morbidity of all new cancer cases, with more than 1.8 million new cases, and the second in mortality, with more than 860,000 deaths (4). CRC has a genetic susceptibility syndrome, but this condition accounts for only a small portion of CRC cases (5). According to the analysis of twin and family studies, the heritability of CRC is only 12–35%. The relatively low heritability level of CRC reflects the importance of the environment, that is, the environment plays a greater role in causing sporadic CRC (6). Cancer prevention is one of the most significant priorities in public health (7). Epidemiological studies on the relationship between dietary habits and disease risk have shown that diet has a direct impact on public health (8). Much effort has been made to assess the preventive chemical effects of natural products in the past few decades. Polyphenols have attracted much attention due to their advantages of few side effects, wide availability, and low toxicity (9). Polyphenols are widely found in the plant kingdom. They are considered potentially useful for anti-inflammatory and anti-tumor drugs, which may be one of the good candidate drugs for cancer prevention and treatment on account of affecting the disease process of cancer in many ways (10, 11). Hence, polyphenols are considered as potential molecular sources for the treatment of various malignant tumors.

## Health-Related Properties of Polyphenols

Polyphenols are phytochemicals existing in plants, widely found in tea, vegetables, soft fruits, and wine, which can regulate intestinal microecological stability and reduce cancer risk (12–14). Although symptoms cannot be described in their absence, *in vitro* and animal model studies have shown that polyphenols have a wide range of pharmacological and therapeutic properties, including anticancer (13), anti-inflammatory (15), antioxidant (16), and vascular protective properties (17). The evidence revealed that epigallocatechin-3-gallate (EGCG) suppressed the growth of melanoma cells by activating 67-kDa laminin receptor (67LR) signaling pathway. That is, 67LR participates in the upregulation of miRNA let-7b expression induced by EGCG through cAMP/protein kinase A (PKA)/protein phosphatase 2A (PP2A) signaling pathway, while the upregulation of let-7b results in the downregulation of a high mobility group A2 (HMGA2), the target gene related to tumor development (18). Olive oil polyphenols represented the pro-oxidative and proinflammatory effects undergoing the representative mixture of oxysterols, while Caco-2 completely differentiates into intestinal epithelial-like cells. In addition, olive oil polyphenols could directly regulate the phosphorylation of p38 and JNK1/2 and the activation of NF-κB after phosphorylation of IκB and maintain the level of NO with that of the control group by inhibiting iNOS induction (19). Polyphenol-rich plum could prevent weight gain and increase the proportion of high-density lipoprotein cholesterol and total cholesterol in plasma. In addition, it could reduce the level of angiotensin II in plasma and its receptor Agtr1 in heart tissue, which reveals that polyphenols may be a receptor-γ agonist activated by peroxisome proliferators, and these results suggest that polyphenol-rich plum may possess the properties of myocardial protection (20). The study showed that chlorogenic acid stimulates the expression of IFN-γ mRNA and increases the number of IFN- γ^+^ CD4^+^ cells in mouse lymphoid aggregation cells, while the number of cells (IFN- γ^+^ CD4^+^, IFN-γ^+^ CD49b^+^, and IL-12^+^ CD11b^+^) in mouse spleen increased significantly, which indicated that polyphenols had a certain stimulating effect on the cellular immune system of mice (21). Table 1 shows the health properties of a variety of polyphenols from different sources. Diet composition and habits are closely related to the occurrence of cancer. The study has shown that green tea polyphenols have a protective effect on female CRC patients, and there is a significant negative correlation between the daily intake of 1 cup of tea and the risk of CRC (30). In addition, the study of F344 rat model treated with n azoxymethane showed that polyphenon E reduced tumor diversity and tumor size while reducing the nuclear expression of β-catenin, inducing apoptosis, and increasing RXRα, β, and γ expression levels in adenocarcinoma (26). Due to the fewer side effects and low toxicity of polyphenols, the biological activity of polyphenols has become a hot topic in many research fields over the years (31).

**Table 1:** The health-related properties and mechanisms of polyphenols.

**References**	**Polyphenols**	**Model**	**Health-related properties**	**The mechanisms**
(22)	*Dendrobium* polyphenols	db/db Mice	Anti-diabetic, anti-inflammatory, antioxidant	Prevented weight gain; decreased the level of glucose and lipoprotein cholesterol; enhanced insulin level; ameliorated the progress of fatty liver and DN; decreased the concentration of MDA; improved the level of MDA, SOD, CAT, and GSH; reduced IL-6 and TNF-α; and enhanced the proportion of *Bacteroidetes* to *Firmicutes*
(23)	Citrus-extract polyphenols	RAW264.7 cells	Anti-inflammatory	Reduced the level of TNF-α, NO, IL-6, and TNF-α; lowered the NFκB in protein expression level; and increased adiponectin concentration
(24)	Resveratrol	Sprague-Dawley CD rats	Prevents mammary cancer	Reduced the proliferation of cells in the structure of the terminal duct of the breast to reduce carcinogenic damage and increased the number of apoptotic cells in the terminal bud epithelial cells of the mammary gland compared with the control group
(25)	Seaweed-polyphenols	PC-CSCs	Prevents pancreatic cancer	Reduced stem-cell transcriptional machinery regulated completely SOX2, OCT3/4, Nanog, LIF, CD44, PIK3R1, N-Cadherin, and E-Cadherin reduced by FIR
(26)	Green tea polyphenols	F344 Rats	Inhibits colorectal tumorigenesis	Reduced tumor diversity and tumor size; suppressed the level of leukotriene B4, proinflammatory eicosanoids and prostaglandin E2 in plasma; decreased the nuclear expression of β-catenin; induced cell apoptosis; and increased the expression level of RXRα, β, and γ in adenocarcinoma
(27)	Polyphenol from foxtail millet bran	HCT-8/Fu cells	Prevents CRC	Suppressed cell proliferation to increase the sensitivity of chemotherapy drugs; facilitated cell apoptosis and promoted the accumulation of Rh-123 in HCT-8/Fu cells; and reduced protein expression, such as MRP1, BCRP, and P-gp
(28)	Resveratrol and curcumin	DLD-1 and Caco-2	Prevents CRC	The regulating effect of the combination of resveratrol and curcumin on apoptosis genes, such as PMAIP1, bid, zmat3, CASP3, CASP7, and FAS in more than a single use
(1)	Plant-derived polyphenols	HT-29 CRC cells	Prevents CRC	Inhibited the growth of HT-29 CRC cells; reduced the expression of bcl-2 by suppressing the activation of NFκB
(29)	Tea polyphenols	SW480 cells and HT-29	Prevents CRC	Suppressed the gene expression of JAG1, MAFA, HES1, MT2A, BAX, and p38 genes relative to the control

*DN, diabetic nephropathy; MDA, malondialdehyde; SOD, superoxide dismutase; CAT, catalase; GSH, glutathione; PC-CSCs, pancreatic cancer—cancer stem cells; Rh-123, rhodamine-123; MRP1, multi-drug resistance protein 1; P-gp, P-glycoprotein; BCRP, breast cancer resistance protein; MAFA, transcription factor; HES1, hairy and enhancer of split 1; JAG1, jagged1*.

## The Physiopathology of Colorectal Cancer

**Figure 1:**
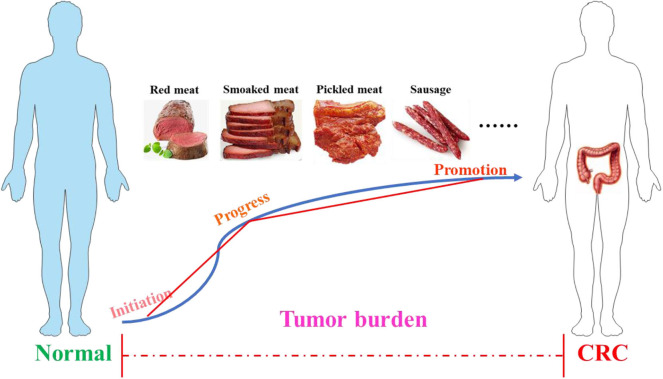
The process of colorectal cancer. The development of cancer includes three stages: initiation, progress, and promotion. Poor dietary habits, such as the long-term consumption of processed meat (smoked meat, picked meat, sausage, etc.) and red meat, may accelerate tumor burden and eventually induce CRC. CRC, colorectal cancer.

The colon is a part of the digestive system and has a complex three-dimensional structure, and the length of the human colon is about 100–150 cm (32, 33). Its main function is to absorb the water and electrolyte left after the small intestine washes and excretes the feces. In terms of deconstruction, the colon begins with the cecum, followed by the colon, transverse colon, descending colon, sigmoid colon, and rectum. This organ is covered by double outer muscles and smooth muscle cells (34). In the mass, the development of cancer can be divided into three stages: initiation, progress, and promotion (35), as shown in Figure 1. The initial stage of CRC starts from the normal mucosa, and the cell replication is indiscriminately disordered and shows abnormal proliferation with the formation of enlarged crypt clusters (36, 37). If there is no appropriate intervention treatment in the development process, the adenoma will expand into CRC, which can spread in the body through metastasis (36). The development of CRC is a multi-step process that follows an adenoma-cancer sequence and has a background of genomic instability (38). CRC shares a number of common molecular characteristics, including microsatellite instability, chromosomal instability, and epigenetic silencing through the CpG island methylated phenotype (39). According to the unique genetic, pathological, and clinical characteristics of these pathways, these can be used for the molecular classification of CRC and comprehensive analysis of tumors to improve the diagnosis, treatment, and prognosis of the disease according to the unused conditions (39). According to the evidence provided by the World Cancer Research Foundation, processed foods, such as those smoked, pickled, or preserved with chemical preservatives, and red meat will increase the possibility of inducing CRC. In contrast, fiber-rich foods can reduce the risk of CRC (40, 41).

## The Transformation and Bioavailability of Polyphenols in the Intestine

Metabolism is a key step in the digestion and absorption of nutrients and further participation in various physiological activities or responses in the host, as well as a key area of interaction between the host and the microbiome (42, 43). Intestinal microbes can metabolize a variety of dietary nutrients, such as indigestible carbohydrates and host metabolites (2). The relationship between dietary polyphenols and the prevention of human malignancies has been a research hotspot in the past few years. One of the reasons for investigating these compounds is that they have a protective effect on CRC (44). In terms of extraction techniques, it can be divided into extractable polyphenols (EPP) and non-extractable polyphenols (NEPP) (45). EPP includes low molecular weight compounds from monomers to decamers, which are soluble in organic solvents, such as methanol, ethanol, and ethyl acetate (46). EPP has a wide range of chemical structures, including hydrolyzed tannins, flavonoids, benzoic acid, hydroxycinnamic acid, stilbene, and others (46). EPP dissolve in the stomach and small intestine, where they can be absorbed at least partially through the small intestinal mucosa, which in turn produces metabolic and systemic effects. For example, dietary extractable proanthocyanidins (EPA) is partially hydrolyzed into epicatechin in the small intestine and absorbed (47, 48). Then a part is widely conjugated in the liver (49), while the other part, together with dietary fiber and indigestible substrates, reaches the colon where the intestinal flora catabolizes to produce metabolites, such as phenylacetic acid, phenylpropionic acid, and phenylbutyric acid (50, 51).

Since NEPP reach the colon almost intact, which is the primary site of their metabolic transformation, it is clear that their primary health trait is gastrointestinal health (52, 53). NEPP are a concentrated tannin and hydrolyzable phenolic high molecular weight compound or polyphenols related to fiber and protein in the diet, which remain in water or organic extracts (54). After being ingested, NEPP are not released from the food matrix through chewing, the acidic environment in the stomach, or the action of digestive enzymes (52). NEPP are not bio-accessible in the small intestine. They reach the colon from the gastrointestinal tract as an insoluble substrate, where they release single polyphenols and different bioavailable metabolites through the workings of the bacterial community in the colon (46). In other words, NEPP can provide absorbable and bioactive metabolites for the intestinal tract once they are fermented by colonic microorganisms. The study showed that 10 ml/kg of proanthocyanidins-rich dietary fiber could induce changes in the expression of tumor suppressor genes and proto-oncogenes genes and affect lipid synthesis, energy metabolism, cell cycle, and apoptosis, which suggested that polyphenols might help reduce the risk of CRC (55).

## Polyphenols Participated in Colorectal Cancer

As a source of biological compounds, natural products have a great potential value because they can reduce/inhibit the risk and development of cancer, which is very useful for medical applications (56). CRC is caused by a series of pathological changes in the transformation of the normal colonic epithelium into invasive carcinoma. The development of CRC spans a multi-step process of 10–15 years, which provides opportunities for its early detection and prevention. Dietary polyphenols have been shown to have anticancer effects by affecting epigenetics (57), inflammation (58), mRNA expression (59–61), and gut microbes (14). The continuous discovery and mining of polyphenols have opened up new directions for the prevention and treatment of cancer. There are now polyphenols and derivatives used in clinical anti-colorectal cancer (62). For instance, silymarin could inhibit Wnt signal transduction in CRC cells to reduce the expression of hydro-catenin and TCF4, thus achieving the apoptosis and proliferation effects on cancer cells. Studies have revealed that EGCG (63), resveratrol (64), and curcumin (65) possess the properties of apoptosis, anti-proliferation, anti-angiogenesis, and cell cycle arrest in the development of CRC.

### Epigenetic Modifications

Traditionally, cancer is regarded as a series of diseases driven by progressive genetic abnormalities, including tumor mutations (suppressor genes and carcinogenic genes), and chromosomal abnormalities (66). However, cancer is also a disease driven by “epigenetic changes,” and they are derived from normal cells, which mediate mechanisms that do not affect the original DNA sequence but regulate heritable changes in gene activity through meiosis and mitosis (67, 68). Epigenetic changes include DNA methylation, histone modification, microRNA, and nucleosome positioning (69). Epigenetic driver genes are thought to be involved in the early stages of the tumor, and DNA methylation changes are a hallmark of CRC (70).

#### DNA Methylation

Abnormal DNA methylation is often detected in gastrointestinal tumors and may therefore be used for the screening, diagnosis, prognosis, and prediction of colorectal cancer (71). DNA methylation is a eukaryotic genomic modification event that occurs at the fifth carbon site of the cytosine residue in CpG dinucleotide, which is essential for mammalian development. Polyphenols in the diet may reduce the risk of colon cancer by altering DNA methylation. EGCG, one of the major polyphenols in green tea, has been shown to inhibit DNA methyltransferase (DNMT) activity and to reactivate methylation-silencing genes in cancer cells. In addition, EGCG can activate methylation-silencing genes in a variety of cancer cells, such as HT-29, KYSE 150, and PC3, indicating that EGCG may prevent cancer by reversing the silencing of related genes (72). The gallic acid portion of the D ring in EGCG can interact with the cytosine active site on the DNMT enzyme, which may be one of the reasons EGCG can be an effective DNMT inhibitor (57). Moreover, the combined application of EGCG and sodium butyrate promoted colorectal cancer cell apoptosis and induced cell cycle arrest and DNA damage (73). Curcumin-induced DNA methylation in CRC cell lines (HCT116, HT29, and RKO) was time-dependent, and curcumin treatment results in methylation changes at partial methylation sites rather than CpG sites that are completely methylated (74). Polyphenols inhibit DNA methylation and DNMT activity in two ways: reducing intracellular S-adenosylmethionine (SAM) concentration and non-competitively inhibiting DNMT activity or inserting DNMT binding vesicles to form a competitive inhibition (75).

#### miRNA

MiRNAs are a new class of small molecule ncRNAs that can be combined by miRNA targets of various signal pathways, usually as a modulator of gene expression (76). MiRNA is dysregulated in cancer cells through the epigenetic mechanism in cancer cells/colonic tumor tissue—overexpressed and underexpressed miRNA (77, 78), suggesting that miRNAs are associated with cancer and may have a vital role in diagnosis, prediction, and as therapeutic targets for CRC (79). There is increasing evidence that polyphenols play an anti-tumor role by regulating miRNA and its target protein in different cancer cells. The study demonstrated that resveratrol had protective properties on colon cancer SW480. The results showed that resveratrol treatment reduced the key effector transforming growth factor β (TGF-β) signal pathway, tumor suppressor PTEN and PDCD4, and Dicer-1, which were responsible for the enzyme process of transforming pre-miRNA into mature miRNA, while enhancing the expression of miR-663, a tumor suppressor microRNA targeting TGF β 1 transcript (80). Moreover, the combination of resveratrol and quercetin (RQ) reduced the production of ROS and improved the antioxidant effect of in the HT-29 cell line. In addition, RQ reduced the expression of Sp1, Sp3, and Sp4 mRNA and reduced microRNA-27a (miR-27a) and the induced Sp-inhibitor zinc finger protein ZBTB10, which indicated that the interaction between RQ and miR-27a- ZBTB10 axis played a role in Sp down-regulation. These indicated that polyphenols could be used as natural anticancer agents (81).

### Intestinal Microbe

Intestinal microbes play a key role in the integration of environmental factors with host physiology and metabolism, which may affect the occurrence and development of CRC through the changes of immune and metabolic signals mediated by metabolism, including the balance of intestinal cell proliferation and death, and the changes of host metabolic activity (82, 83). CRC is closely related to microbial changes near the mucosa where the tumor is located, and part of this ecological disorder is characterized by the expansion of bacterial taxa, while the dominant species in the development of CRC is still unclear (84, 85). Although the available epidemiological evidence is limited, relatively consistent research data indicates that the number of butyrate-producing bacteria in CRC has decreased while *Fusobacterium nucleatum* (Fn) and *Bacteroides fragilis* have increased (86, 87). Moreover, a retrospective analysis of 13,096 adult bacteremia without a history of cancer indicates that the late diagnosis of CRC may be related to *Bacteroides fragilis* and *Streptococcus gallolyticus*, which may be related to intestinal disorders and barrier dysfunction that cause these bacteria to enter the bloodstream (88). As mentioned above, NEPP have bioavailability in the colon, where polyphenols and gut microbes form a two-way interaction in which polyphenols regulate the composition and diversity of intestinal microbes. At the same time, intestinal microorganisms decompose the ingested polyphenols and then release more active and easily absorbed metabolites than natural polyphenols, which may provide more possibilities for preventing CRC (89, 90). The correlation analysis of the gram-negative anaerobic commensal bacteria Fn and human CRC genome showed that in 99 CRC patients, fluorescence *in situ* hybridization was used to connect CRC and Fn amplification. It was found that the Clostridium sequence was associated with lymph node metastasis (91, 92). Tea polyphenols have different degrees of antibacterial activity, which may hinder the bacterial cell membrane and the chelation of iron. The study showed that EGCG and theaflavin could reduce the expression of two virulence factors (hemolysis and hydrogen sulfide) expressed by FN and inhibit the adhesion of FN to oral epithelial cells and matrix proteins (93). Mucus-associated *Escherichia coli* (*E. coli*) is more common in CRC tissues, and their abundance is associated with tumor stage and prognosis (94). Pathogenic *E. coli* producing pathogenic colibactin is more common in terminal illness, and the CRC-related *E. coli* could significantly increase the number of polyps in *Apc*^Min/+^ mice, indicating that *E. coli* may promote the occurrence of tumors (95). Red wine polyphenols could be dose-dependent to prevent intestinal cytotoxicity to HT-29 caused by *E. coli* 270 (96). How microbial diversity suppresses tumorigenesis and which species or their interactions are associated with CRC development remain to be further explored. In addition, it is challenging to explore the effect of the abundance of specific strains in commensal bacteria on the development of CRC experimentally (87).

## Other Potential Roles of Polyphenols on Intestinal Inflammation

The intestine is a multi-functional organ whose functions include digestion/absorption, barrier function, and recognition of external stimuli and signal transduction (97). Chronic inflammation occurs when the gut is constantly damaged by external stress (such as food, bacteria, and environmental chemicals). Inflammation is a non-specific immune response that protects the body from myriad biological and chemical threats in the surrounding environment, relying on the dense cellular and molecular control mechanisms of the intestinal mucosa (98). Polyphenols may play an anticancer role by regulating inflammatory pathways through key signal transduction pathways and thus affect the course of the disease. The key signaling pathways include NFκB and signal transducer and activator of transcription (STAT3), as well as phosphatidylinositol 3-kinase (PI3K) and cyclo-oxygenase (COX) (99). Curcumin mediates its chemotherapy by regulating NFκB transcription factor expression, inhibiting NFκB-regulated gene products, such as COX-2, adhesion molecule, matrix metalloproteinases (MMPs), iNOS, Bcl-2, and TNF, and regulating cyclin expression (cyclin D1 and p21) (100). The upregulation of STAT3 can attract chemokines from immune and inflammatory cells to induce tumor-related inflammation (101). EGCG could inhibit the phosphorylation of STAT3 (102), while resveratrol inhibits IL-6-induced intercellular cell adhesion molecule-1 (ICAM-1) gene expression by interfering with STAT3 phosphorylation (103). The PI3K signaling pathway plays a vital role in tumorigenesis, which includes inhibiting apoptosis, increasing cell proliferation and growth, and reducing cell cycle arrest (104, 105). The study has shown that curcumin inhibited the expression of oncogene MDM2 through the PI3K signaling pathway, which is related to cell survival and proliferation (106). Moreover, cyclo-oxygenase-2 (COX2) is an inducer of prostaglandin synthesis, and its tumor-promoting effect is mediated by its main end product, prostaglandin PGE2 (107). COX2 has displayed overexpression during the development of CRCs. The study of pomegranate extract could significantly downregulate the expression of COX-2 protein and the constitutive expression and phosphorylation of NF-kB p65 in HT-29 cells (108).

## Concluding Remarks

Due to uncontrolled cell division in cancer, the development of cancer is related to many factors, including diagnosis, drug-induced cytotoxicity, chemotherapy resistance, and prognosis. The development cycle is long, affecting a large number of people. Inhibiting DNMT activity and regulating mRNA changes and intestinal changes through natural compounds may be powerful approaches to cancer prevention by regulating multiple cell functions to destroy multi-stage cancer diseases, thereby mediating multiple anticancer pathways. Polyphenols are widely found in food and beverages, and the combination of polyphenols and traditional drugs can improve therapeutic effects, reduce the drug resistance of tumor cells, and reduce the toxicity of chemotherapy drugs. The influence of polyphenols on the intestinal microbiota has been widely recognized, but their changes and mechanism of action in the progress of CRC need a lot of research and supplement. Substantial evidence proves that polyphenols are among the best choices for the prevention and treatment of CRC. On the concentration and bioavailability of polyphenols, whether a moderate-to-high dose will have a beneficial effect on health without affecting gut barrier integrity and gut microbe remains unclear. Whether the rich and complex polyphenols in the daily diet will affect the therapeutic effect of polyphenols is also unclear. That may be further elucidated with the development of techniques and research in the field of omics.

## Author Contributions

SD and SX: writing—original draft preparation. JF and HJ: writing—review and editing. All authors contributed to manuscript revision, read, and approved the submitted version.

## Conflict of Interest

The authors declare that the research was conducted in the absence of any commercial or financial relationships that could be construed as a potential conflict of interest.

## References

[1] Priego S Feddi F Ferrer P Mena S Benlloch M Ortega A. Natural polyphenols facilitate elimination of HT-29 colorectal cancer xenografts by chemoradiotherapy: a Bcl-2- and superoxide dismutase 2-dependent mechanism. Mol Cancer Ther. (2008) 7:3330–42. 10.1158/1535-7163.MCT-08-0363 18852136

[2] Wong SH Yu J. Gut microbiota in colorectal cancer: mechanisms of action and clinical applications. Nat Rev Gastroenterol Hepatol. (2019) 16:690–704. 10.1038/s41575-019-0209-8 31554963

[3] Lichtenstein P Holm NV Verkasalo PK Iliadou A Kaprio J Koskenvuo M. Environmental and heritable factors in the causation of cancer–analyses of cohorts of twins from Sweden, Denmark, and Finland. N Engl J Med. (2000) 343:78–85. 10.1056/NEJM200007133430201 10891514

[4] Bray F Ferlay J Soerjomataram I Siegel RL Torre LA Jemal A. Global cancer statistics 2018: GLOBOCAN estimates of incidence and mortality worldwide for 36 cancers in 185 countries. CA Cancer J Clin. (2018) 68:394–424. 10.3322/caac.21492 30207593

[5] Foulkes WD. Inherited susceptibility to common cancers. N Engl J Med. (2008) 359:2143–53. 10.1056/NEJMra0802968 19005198

[6] Czene K Lichtenstein P Hemminki K. Environmental and heritable causes of cancer among 9.6 million individuals in the Swedish family-cancer database. Int J Cancer. (2002) 99:260–6. 10.1002/ijc.10332 11979442

[7] Hostanska K Jurgenliemk G Abel G Nahrstedt A Saller R. Willow bark extract (BNO1455) and its fractions suppress growth and induce apoptosis in human colon and lung cancer cells. Cancer Detect Prev. (2007) 31:129–39. 10.1016/j.cdp.2007.03.001 17418981

[8] Espin JC Garcia-Conesa MT Tomas-Barberan FA. Nutraceuticals: facts and fiction. Phytochemistry. (2007) 68:2986–3008. 10.1016/j.phytochem.2007.09.014 17976666

[9] D'Archivio M Santangelo C Scazzocchio B Varì R Filesi C Masella R. Modulatory effects of polyphenols on apoptosis induction: relevance for cancer prevention. Int J Mol Sci. (2008) 9:213–28. 10.3390/ijms9030213 19325744 PMC2635670

[10] Yang CS Wang X Lu G Picinich SC. Cancer prevention by tea: animal studies, molecular mechanisms and human relevance. Nat Rev Cancer. (2009) 9:429–39. 10.1038/nrc2641 19472429 PMC2829848

[11] Elshaer M Chen Y Wang XJ Tang X. Resveratrol: an overview of its anti-cancer mechanisms. Life Sci. (2018) 207:340–9. 10.1016/j.lfs.2018.06.028 29959028

[12] Duthie SJ. Berry phytochemicals, genomic stability and cancer: evidence for chemoprotection at several stages in the carcinogenic process. Mol Nutr Food Res. (2007) 51:665–74. 10.1002/mnfr.200600257 17487926

[13] Han Y Huang M Li L Cai X Gao Z Li F. Non-extractable polyphenols from cranberries: potential anti-inflammation and anti-colon-cancer agents. Food Func. (2019) 10:7714–23. 10.1039/C9FO01536A 31750473

[14] Naumovski N Panagiotakos DB D'Cunha NM. Untangling the 2-way relationship between red wine polyphenols and gut microbiota. Gastroenterology. (2020) 158:48–51. 10.1053/j.gastro.2019.10.015 31628899

[15] Yahfoufi N Alsadi N Jambi M Matar C. The immunomodulatory and anti-inflammatory role of polyphenols. Nutrients. (2018) 10:1618. 10.3390/nu10111618 30400131 PMC6266803

[16] Goni I Hernandez-Galiot A Intake of nutrient and non-nutrient dietary antioxidants. Contribution of macromolecular antioxidant polyphenols in an elderly mediterranean population. Nutrients. (2019) 11:2165 10.3390/nu11092165 PMC6769609 31509947

[17] Adriouch S Lampure A Nechba A Baudry J Assmann K Kesse-Guyot E. Prospective association between total and specific dietary polyphenol intakes and cardiovascular disease risk in the Nutrinet-Santé French cohort. Nutrients. (2018) 10:1587. 10.3390/nu10111587 30380657 PMC6266343

[18] Yamada S Tsukamoto S Huang Y Makio A Kumazoe M Yamashita S. Epigallocatechin-3-O-gallate up-regulates microRNA-let-7b expression by activating 67-kDa laminin receptor signaling in melanoma cells. Sci Rep. (2016) 6:19225. 10.1038/srep19225 26754091 PMC4709792

[19] Serra G Incani A Serreli G Porru L Melis MP Tuberoso CIG. Olive oil polyphenols reduce oxysterols -induced redox imbalance and pro-inflammatory response in intestinal cells. Redox Biol. (2018) 17:348–54. 10.1016/j.redox.2018.05.006 29793168 PMC6007813

[20] Noratto G Martino HS Simbo S Byrne D Mertens-Talcott SU. Consumption of polyphenol-rich peach and plum juice prevents risk factors for obesity-related metabolic disorders and cardiovascular disease in zucker rats. J Nutr Biochem. (2015) 26:633–41. 10.1016/j.jnutbio.2014.12.014 25801980

[21] Karasawa K Uzuhashi Y Hirota M Otani H. A matured fruit extract of date palm tree (*Phoenix dactylifera* L.) stimulates the cellular immune system in mice. J Agric Food Chem. (2011) 59:11287–93. 10.1021/jf2029225 21936496

[22] Li XW Chen HP He YY Chen WL Chen JW Gao L. Effects of rich-polyphenols extract of dendrobium loddigesii on anti-diabetic, anti-inflammatory, anti-oxidant, and gut microbiota modulation in db/db mice. Molecules. (2018) 23:3245. 10.3390/molecules23123245 30544624 PMC6320866

[23] Nakajima VM Moala T Caria C Moura CS Amaya-Farfan J Gambero A. Biotransformed citrus extract as a source of anti-inflammatory polyphenols: effects in macrophages and adipocytes. Food Res Int. (2017) 97:37–44. 10.1016/j.foodres.2017.03.034 28578062

[24] Li Y Zhang T. Targeting cancer stem cells by curcumin and clinical applications. Cancer Lett. (2014) 346:197–205. 10.1016/j.canlet.2014.01.012 24463298

[25] Aravindan S Ramraj SK Somasundaram ST Herman TS Aravindan N. Polyphenols from marine brown algae target radiotherapy-coordinated EMT and stemness-maintenance in residual pancreatic cancer. Stem Cell Res Ther. (2015) 6:182. 10.1186/s13287-015-0173-3 26395574 PMC4578749

[26] Hao X Xiao H Ju J Lee MJ Lambert JD Yang CS. Green tea polyphenols inhibit colorectal tumorigenesis in azoxymethane-treated F344 rats. Nutr Cancer. (2017) 69:623–31. 10.1080/01635581.2017.1295088 28323438

[27] Lu Y Shan S Li H Shi J Zhang X Li Z. Reversal effects of bound polyphenol from foxtail millet bran on multidrug resistance in human HCT-8/Fu colorectal cancer cell. J Agric Food Chem. (2018) 66:5190–9. 10.1021/acs.jafc.8b01659 29730933

[28] Gavrilas LI Cruceriu D Ionescu C Miere D Balacescu O. Pro-apoptotic genes as new targets for single and combinatorial treatments with resveratrol and curcumin in colorectal cancer. Food Func. (2019) 10:3717–26. 10.1039/C9FO01014A 31169275

[29] Jin H Tan X Liu X Ding Y. The study of effect of tea polyphenols on microsatellite instability colorectal cancer and its molecular mechanism. Int J Colorectal Dis. (2010) 25:1407–15. 10.1007/s00384-010-1047-x 20730438

[30] Chen Y Wu Y Du M Chu H Zhu L Tong N. An inverse association between tea consumption and colorectal cancer risk. Oncotarget. (2017) 8:37367–76. 10.18632/oncotarget.16959 28454102 PMC5514915

[31] Li D Chen Y Huang Y Zhang L Yang J Xu X Study on the anti-tumor ability of niaowangzhong green tea. J Food Biochem. (2017) 41:e12305 10.1111/jfbc.12305

[32] Ellis H Mahadevan V Anatomy of the caecum, appendix and colon. Surgery. (2014) 32:155–8. 10.1016/j.mpsur.2014.02.001

[33] Ponz de Leon M Di Gregorio C. Pathology of colorectal cancer. Dig Liver Dis. (2001) 33:372–88. 10.1016/S1590-8658(01)80095-5 11432519

[34] Santos IS Ponte BM Boonme P Silva AM Souto EB. Nanoencapsulation of polyphenols for protective effect against colon-rectal cancer. Biotechnol Adv. (2013) 31:514–23. 10.1016/j.biotechadv.2012.08.005 22940401

[35] Shehzad A Wahid F Lee YS. Curcumin in cancer chemoprevention: molecular targets, pharmacokinetics, bioavailability, and clinical trials. Arch Pharm. (2010) 343:489–99. 10.1002/ardp.200900319 20726007

[36] Boghossian S Hawash A. Chemoprevention in colorectal cancer–where we stand and what we have learned from twenty year's experience. Surgeon. (2012) 10:43–52. 10.1016/j.surge.2011.07.003 22129884

[37] Sharma RA Manson MM Gescher A Steward WP. Colorectal cancer chemoprevention: biochemical targets and clinical development of promising agents. Eur J Cancer. (2001) 37:12–22. 10.1016/S0959-8049(00)00326-9 11165125

[38] Collins D Hogan AM Winter DC. Microbial and viral pathogens in colorectal cancer. Lancet Oncol. (2011) 12:504–12. 10.1016/S1470-2045(10)70186-8 21067973

[39] Soreide K Nedrebo BS Knapp JC Glomsaker TB Soreide JA Korner H. Evolving molecular classification by genomic and proteomic biomarkers in colorectal cancer: potential implications for the surgical oncologist. Surg Oncol. (2009) 18:31–50. 10.1016/j.suronc.2008.06.006 18672360

[40] Research WCRFAIfC Continuous Update Project Report. Food, Nutrition, Physical Activity, and the Prevention of Colorectal Cancer. (2011). Available online at: https://www.wcrf.org/sites/default/files/Colorectal-Cancer-2011-Report.pdf

[41] Sobiecki JG Vegetarianism and colorectal cancer risk in a low-selenium environment: effect modification by selenium status? A possible factor contributing to the null results in British vegetarians. Eur J Nutr. (2017) 56:1819–32. 10.1007/s00394-016-1364-0 28191611 PMC5534195

[42] Doll R Peto R. The causes of cancer: quantitative estimates of avoidable risks of cancer in the United States today. J Natl Cancer Inst. (1981) 66:1191–308. 10.1093/jnci/66.6.1192 7017215

[43] Zhang FF Cudhea F Shan Z Michaud DS Imamura F Eom H. Preventable cancer burden associated with poor diet in the United States. JNCI Cancer Spectrum. (2019) 3:pkz034. 10.1093/jncics/pkz034 31360907 PMC6649723

[44] Araujo JR Goncalves P Martel F. Chemopreventive effect of dietary polyphenols in colorectal cancer cell lines. Nutr Res. (2011) 31:77–87. 10.1016/j.nutres.2011.01.006 21419311

[45] Macagnan FT da Silva LP Hecktheuer LH. Dietary fibre: the scientific search for an ideal definition and methodology of analysis, and its physiological importance as a carrier of bioactive compounds. Food Res Int. (2016) 85:144–54. 10.1016/j.foodres.2016.04.032 29544829

[46] Saura-Calixto F. Concept and health-related properties of nonextractable polyphenols: the missing dietary polyphenols. J Agric Food Chem. (2012) 60:11195–200. 10.1021/jf303758j 23095074

[47] Baba S Osakabe N Natsume M Terao J. Absorption and urinary excretion of procyanidin B2 [epicatechin-(4beta-8)-epicatechin] in rats. Free Radical Biol Med. (2002) 33:142–8. 10.1016/S0891-5849(02)00871-7 12086692

[48] Deprez S Mila I Huneau JF Tome D Scalbert A. Transport of proanthocyanidin dimer, trimer, and polymer across monolayers of human intestinal epithelial Caco-2 cells. Antioxid Redox Signal. (2001) 3:957–67. 10.1089/152308601317203503 11813991

[49] Scalbert A Morand C Manach C Remesy C. Absorption and metabolism of polyphenols in the gut and impact on health. Biomed Pharmacother. (2002) 56:276–82. 10.1016/S0753-3322(02)00205-6 12224598

[50] Gonthier MP Donovan JL Texier O Felgines C Remesy C Scalbert A. Metabolism of dietary procyanidins in rats. Free Radical Biol Med. (2003) 35:837–44. 10.1016/S0891-5849(03)00394-0 14556848

[51] Saura-Calixto F Perez-Jimenez J Tourino S Serrano J Fuguet E Torres JL. Proanthocyanidin metabolites associated with dietary fibre from *in vitro* colonic fermentation and proanthocyanidin metabolites in human plasma. Mol Nutr Food Res. (2010) 54:939–46. 10.1002/mnfr.200900276 20087856

[52] Perez-Jimenez J Diaz-Rubio ME Saura-Calixto F. Non-extractable polyphenols, a major dietary antioxidant: occurrence, metabolic fate and health effects. Nutr Res Rev. (2013) 26:118–29. 10.1017/S0954422413000097 23930641

[53] Larrosa M Luceri C Vivoli E Pagliuca C Lodovici M Moneti G. Polyphenol metabolites from colonic microbiota exert anti-inflammatory activity on different inflammation models. Mol Nutr Food Res. (2009) 53:1044–54. 10.1002/mnfr.200800446 19557820

[54] Perez-Jimenez J Saura-Calixto F. Literature data may underestimate the actual antioxidant capacity of cereals. J Agric Food Chem. (2005) 53:5036–40. 10.1021/jf050049u 15941353

[55] Lizarraga D Vinardell MP Noe V van Delft JH Alcarraz-Vizan G van Breda SG. A lyophilized red grape pomace containing proanthocyanidin-rich dietary fiber induces genetic and metabolic alterations in colon mucosa of female C57BL/6J mice. J Nutr. (2011) 141:1597–604. 10.3945/jn.110.133199 21775529

[56] Nair HB Sung B Yadav VR Kannappan R Chaturvedi MM Aggarwal BB. Delivery of antiinflammatory nutraceuticals by nanoparticles for the prevention and treatment of cancer. Biochem Pharmacol. (2010) 80:1833–43. 10.1016/j.bcp.2010.07.021 20654584 PMC2974020

[57] Duthie SJ. Epigenetic modifications and human pathologies: cancer and CVD. Proc Nutr Soc. (2011) 70:47–56. 10.1017/S0029665110003952 21067630

[58] Marzocchella L Fantini M Benvenuto M Masuelli L Tresoldi I Modesti A. Dietary flavonoids: molecular mechanisms of action as anti- inflammatory agents. Recent Patents Inflam Allergy Drug Discov. (2011) 5:200–20. 10.2174/187221311797264937 21827399

[59] Yi J Li S Wang C Cao N Qu H Cheng C. Potential applications of polyphenols on main ncRNAs regulations as novel therapeutic strategy for cancer. Biomed Pharmacothe. (2019) 113:108703. 10.1016/j.biopha.2019.108703 30870719

[60] Li W Yang W Liu Y Chen S Chin S Qi X. MicroRNA-378 enhances inhibitory effect of curcumin on glioblastoma. Oncotarget. (2017) 8:73938–46. 10.18632/oncotarget.17881 29088758 PMC5650313

[61] Bi Y Shen W Min M Liu Y. MicroRNA-7 functions as a tumor-suppressor gene by regulating ILF2 in pancreatic carcinoma. Int J Mol Med. (2017) 39:900–6. 10.3892/ijmm.2017.2894 28259961 PMC5360436

[62] Huang XM Yang ZJ Xie Q Zhang ZK Zhang H Ma JY. Natural products for treating colorectal cancer: a mechanistic review. Biomed Pharmacother. (2019) 117:109142. 10.1016/j.biopha.2019.109142 31238258

[63] Stingl JC Ettrich T Muche R Wiedom M Brockmoller J Seeringer A. Protocol for minimizing the risk of metachronous adenomas of the colorectum with green tea extract (MIRACLE): a randomised controlled trial of green tea extract versus placebo for nutriprevention of metachronous colon adenomas in the elderly population. BMC Cancer. (2011) 11:360. 10.1186/1471-2407-11-360 21851602 PMC3176243

[64] Fuggetta MP Lanzilli G Tricarico M Cottarelli A Falchetti R Ravagnan G. Effect of resveratrol on proliferation and telomerase activity of human colon cancer cells *in vitro*. J Exp Clin Cancer Res. (2006) 25:189–93. 10.3892/ijo.28.3.641 16918129

[65] Li YH Niu YB Sun Y Zhang F Liu CX Fan L. Role of phytochemicals in colorectal cancer prevention. World J Gastroenterol. (2015) 21:9262–72. 10.3748/wjg.v21.i31.9262 26309353 PMC4541379

[66] Kluth M Jung S Habib O Eshagzaiy M Heinl A Amschler N. Deletion lengthening at chromosomes 6q and 16q targets multiple tumor suppressor genes and is associated with an increasingly poor prognosis in prostate cancer. Oncotarget. (2017) 8:108923–35. 10.18632/oncotarget.22408 29312579 PMC5752492

[67] Feinberg AP Ohlsson R Henikoff S. The epigenetic progenitor origin of human cancer. Nat Rev Genet. (2006) 7:21–33. 10.1038/nrg1748 16369569

[68] Choong MK Tsafnat G. Genetic and epigenetic biomarkers of colorectal cancer. Clin Gastroenterol Hepatol. (2012) 10:9–15. 10.1016/j.cgh.2011.04.020 21635968

[69] Cai L. Cadmium and its epigenetic effects. Curr Med Chem. (2012) 19:2611–20. 10.2174/092986712800492913 22471978

[70] Weisenberger DJ Liang G Lenz HJ. DNA methylation aberrancies delineate clinically distinct subsets of colorectal cancer and provide novel targets for epigenetic therapies. Oncogene. (2018) 37:566–77. 10.1038/onc.2017.374 28991233 PMC7491233

[71] Hashimoto Y Zumwalt TJ Goel A. DNA methylation patterns as noninvasive biomarkers and targets of epigenetic therapies in colorectal cancer. Epigenomics. (2016) 8:685–703. 10.2217/epi-2015-0013 27102979 PMC4928499

[72] Fang MZ Wang Y Ai N Hou Z Sun Y Lu H. Tea polyphenol (-)-epigallocatechin-3-gallate inhibits DNA methyltransferase and reactivates methylation-silenced genes in cancer cell lines. Cancer Res. (2003) 63:7563–70. 14633667

[73] Saldanha SN Kala R Tollefsbol TO. Molecular mechanisms for inhibition of colon cancer cells by combined epigenetic-modulating epigallocatechin gallate and sodium butyrate. Exp Cell Res. (2014) 324:40–53. 10.1016/j.yexcr.2014.01.024 24518414 PMC4043227

[74] Link A Balaguer F Shen Y Lozano JJ Leung HC Boland CR. Curcumin modulates DNA methylation in colorectal cancer cells. PLoS ONE. (2013) 8:e57709. 10.1371/journal.pone.0057709 23460897 PMC3584082

[75] Fang M Chen D Yang CS. Dietary polyphenols may affect DNA methylation. J Nutr. (2007) 137(Suppl. 1):223–8s. 10.1093/jn/137.1.223S 17182830

[76] Jafri MA Al-Qahtani MH Shay JW. Role of miRNAs in human cancer metastasis: Implications for therapeutic intervention. Semin Cancer Biol. (2017) 44:117–31. 10.1016/j.semcancer.2017.02.004 28188828

[77] Schetter AJ Leung SY Sohn JJ Zanetti KA Bowman ED Yanaihara N. MicroRNA expression profiles associated with prognosis and therapeutic outcome in colon adenocarcinoma. JAMA. (2008) 299:425–36. 10.1001/jama.299.4.425 18230780 PMC2614237

[78] Slaby O Svoboda M Fabian P Smerdova T Knoflickova D Bednarikova M. Altered expression of miR-21, miR-31, miR-143 and miR-145 is related to clinicopathologic features of colorectal cancer. Oncology. (2007) 72:397–402. 10.1159/000113489 18196926

[79] Gu HF Mao XY Du M. Prevention of breast cancer by dietary polyphenols-role of cancer stem cells. Crit Rev Food Sci Nutr. (2020) 60:810–25. 10.1080/10408398.2018.1551778 30632783 PMC6625949

[80] Tili E Michaille JJ Alder H Volinia S Delmas D Latruffe N. Resveratrol modulates the levels of microRNAs targeting genes encoding tumor-suppressors and effectors of TGFbeta signaling pathway in SW480 cells. Biochem Pharmacol. (2010) 80:2057–65. 10.1016/j.bcp.2010.07.003 20637737 PMC3918904

[81] Del Follo-Martinez A Banerjee N Li X Safe S Mertens-Talcott S. Resveratrol and quercetin in combination have anticancer activity in colon cancer cells and repress oncogenic microRNA-27a. Nutr Cancer. (2013) 65:494–504. 10.1080/01635581.2012.725194 23530649

[82] Kau AL Ahern PP Griffin NW Goodman AL Gordon JI. Human nutrition, the gut microbiome and the immune system. Nature. (2011) 474:327–36. 10.1038/nature10213 21677749 PMC3298082

[83] Garrett WS. Cancer and the microbiota. Science. (2015) 348:80–6. 10.1126/science.aaa4972 25838377 PMC5535753

[84] Nakatsu G Li X Zhou H Sheng J Wong SH Wu WK. Gut mucosal microbiome across stages of colorectal carcinogenesis. Nat Commun. (2015) 6:8727. 10.1038/ncomms9727 26515465 PMC4640069

[85] Yazici C Wolf PG Kim H Cross TL Vermillion K Carroll T. Race-dependent association of sulfidogenic bacteria with colorectal cancer. Gut. (2017) 66:1983–94. 10.1136/gutjnl-2016-313321 28153960 PMC5575988

[86] Sears CL Garrett WS. Microbes, microbiota, and colon cancer. Cell Host Microbe. (2014) 15:317–28. 10.1016/j.chom.2014.02.007 24629338 PMC4003880

[87] Tilg H Adolph TE Gerner RR Moschen AR. The intestinal microbiota in colorectal cancer. Cancer Cell. (2018) 33:954–64. 10.1016/j.ccell.2018.03.004 29657127

[88] Kwong TNY Wang X Nakatsu G Chow TC Tipoe T Dai RZW. Association between bacteremia from specific microbes and subsequent diagnosis of colorectal cancer. Gastroenterology. (2018) 155:383–90.e8. 10.1053/j.gastro.2018.04.028 29729257

[89] Cueva C Sanchez-Patan F Monagas M Walton GE Gibson GR Martin-Alvarez PJ. *In vitro* fermentation of grape seed flavan-3-ol fractions by human faecal microbiota: changes in microbial groups and phenolic metabolites. FEMS Microbiol Ecol. (2013) 83:792–805. 10.1111/1574-6941.12037 23121387

[90] Munoz-Gonzalez I Jimenez-Giron A Martin-Alvarez PJ Bartolome B Moreno-Arribas MV. Profiling of microbial-derived phenolic metabolites in human feces after moderate red wine intake. J Agric Food Chem. (2013) 61:9470–9. 10.1021/jf4025135 24010549

[91] Castellarin M Warren RL Freeman JD Dreolini L Krzywinski M Strauss J. *Fusobacterium nucleatum* infection is prevalent in human colorectal carcinoma. Genome Res. (2012) 22:299–306. 10.1101/gr.126516.111 22009989 PMC3266037

[92] Kostic AD Gevers D Pedamallu CS Michaud M Duke F Earl AM. Genomic analysis identifies association of Fusobacterium with colorectal carcinoma. Genome Res. (2012) 22:292–8. 10.1101/gr.126573.111 22009990 PMC3266036

[93] Ben Lagha A Haas B Grenier D. Tea polyphenols inhibit the growth and virulence properties of *Fusobacterium nucleatum*. Sci Rep. (2017) 7:44815. 10.1038/srep44815 28322293 PMC5359671

[94] Cougnoux A Dalmasso G Martinez R Buc E Delmas J Gibold L. Bacterial genotoxin colibactin promotes colon tumour growth by inducing a senescence-associated secretory phenotype. Gut. (2014) 63:1932–42. 10.1136/gutjnl-2013-305257 24658599

[95] Veziant J Gagniere J Jouberton E Bonnin V Sauvanet P Pezet D. Association of colorectal cancer with pathogenic *Escherichia coli*: focus on mechanisms using optical imaging. World J Clin Oncol. (2016) 7:293–301. 10.5306/wjco.v7.i3.293 27298769 PMC4896897

[96] Nunes C Figueiredo R Laranjinha J da Silva GJ. Intestinal cytotoxicity induced by *Escherichia coli* is fully prevented by red wine polyphenol extract: mechanistic insights in epithelial cells. Chem Biol Interact. (2019) 310:108711. 10.1016/j.cbi.2019.06.024 31207224

[97] Martin DA Bolling BW. A review of the efficacy of dietary polyphenols in experimental models of inflammatory bowel diseases. Food Func. (2015) 6:1773–86. 10.1039/C5FO00202H 25986932

[98] Fiocchi C. More answers and more questions in inflammatory bowel disease. Curr Opin Gastroenterol. (2003) 19:325–6. 10.1097/00001574-200307000-00001 15703572

[99] Little CH Combet E McMillan DC Horgan PG Roxburgh CS. The role of dietary polyphenols in the moderation of the inflammatory response in early stage colorectal cancer. Crit Rev Food Sci Nutr. (2017) 57:2310–20. 10.1080/10408398.2014.997866 26066365

[100] Shishodia S Sethi G Aggarwal BB. Curcumin: getting back to the roots. Ann N Y Acad Sci. (2005) 1056:206–17. 10.1196/annals.1352.010 16387689

[101] Yu H Pardoll D Jove R. STATs in cancer inflammation and immunity: a leading role for STAT3. Nat Rev Cancer. (2009) 9:798–809. 10.1038/nrc2734 19851315 PMC4856025

[102] Masuda M Suzui M Weinstein IB. Effects of epigallocatechin-3-gallate on growth, epidermal growth factor receptor signaling pathways, gene expression, and chemosensitivity in human head and neck squamous cell carcinoma cell lines. Clin Cancer Res. (2001) 7:4220–9. 11751523

[103] Wung BS Hsu MC Wu CC Hsieh CW. Resveratrol suppresses IL-6-induced ICAM-1 gene expression in endothelial cells: effects on the inhibition of STAT3 phosphorylation. Life Sci. (2005) 78:389–97. 10.1016/j.lfs.2005.04.052 16150460

[104] Samuels Y Wang Z Bardelli A Silliman N Ptak J Szabo S. High frequency of mutations of the PIK3CA gene in human cancers. Science. (2004) 304:554. 10.1126/science.1096502 15016963

[105] Le NH Franken P Fodde R. Tumour-stroma interactions in colorectal cancer: converging on beta-catenin activation and cancer stemness. Br J Cancer. (2008) 98:1886–93. 10.1038/sj.bjc.6604401 18506144 PMC2441948

[106] Li M Zhang Z Hill DL Wang H Zhang R. Curcumin, a dietary component, has anticancer, chemosensitization, and radiosensitization effects by down-regulating the MDM2 oncogene through the PI3K/mTOR/ETS2 pathway. Cancer Res. (2007) 67:1988–96. 10.1158/0008-5472.CAN-06-3066 17332326

[107] Hull M Langman M. Differential expression of cyclooxygenase 2 in human colorectal cancer. Gut. (2000) 47:154. 10.1136/gut.47.1.154 10917748 PMC1727963

[108] Banerjee N Kim H Talcott S Mertens-Talcott S. Pomegranate polyphenolics suppressed azoxymethane-induced colorectal aberrant crypt foci and inflammation: possible role of miR-126/VCAM-1 and miR-126/PI3K/AKT/mTOR. Carcinogenesis. (2013) 34:2814–22. 10.1093/carcin/bgt295 23996930

